# Contrasting Properties of α7-Selective Orthosteric and Allosteric Agonists Examined on Native Nicotinic Acetylcholine Receptors

**DOI:** 10.1371/journal.pone.0055047

**Published:** 2013-01-29

**Authors:** JasKiran K. Gill, Anna Chatzidaki, Daniel Ursu, Emanuele Sher, Neil S. Millar

**Affiliations:** 1 Department of Neuroscience, Physiology and Pharmacology, University College London, London, United Kingdom; 2 Lilly Research Centre, Eli Lilly & Co. Ltd., Windlesham, Surrey, United Kingdom; University of Sydney, Australia

## Abstract

Subtype-selective ligands are important tools for the pharmacological characterisation of neurotransmitter receptors. This is particularly the case for nicotinic acetylcholine receptors (nAChRs), given the heterogeneity of their subunit composition. In addition to agonists and antagonists that interact with the extracellular orthosteric nAChR binding site, a series of nAChR allosteric modulators have been identified that interact with a distinct transmembrane site. Here we report studies conducted with three pharmacologically distinct nicotinic ligands, an orthosteric agonist (compound B), a positive allosteric modulator (TQS) and an allosteric agonist (4BP-TQS). The primary focus of the work described in this study is to examine the suitability of these compounds for the characterisation of native neuronal receptors (both rat and human). However, initial experiments were conducted on recombinant nAChRs demonstrating the selectivity of these three compounds for α7 nAChRs. In patch-clamp recordings on rat primary hippocampal neurons we found that all these compounds displayed pharmacological properties that mimicked closely those observed on recombinant α7 nAChRs. However, it was not possible to detect functional responses with compound B, an orthosteric agonist, using a fluorescent intracellular calcium assay on either rat hippocampal neurons or with human induced pluripotent stem cell-derived neurons (iCell neurons). This is, presumably, due to the rapid desensitisation of α7 nAChR that is induced by orthosteric agonists. In contrast, clear agonist-evoked responses were observed in fluorescence-based assays with the non-desensitising allosteric agonist 4BP-TQS and also when compound B was co-applied with the non-desensitising positive allosteric modulator TQS. In summary, we have demonstrated the suitability of subtype-selective orthosteric and allosteric ligands for the pharmacological identification and characterisation of native nAChRs and the usefulness of ligands that minimise receptor desensitisation for the characterisation of α7 nAChRs in fluorescence-based assays.

## Introduction

In common with other members of the Cys-loop family of ligand-gated ion channels, nAChRs are pentameric neurotransmitter receptors in which agonists and competitive antagonists bind at a site located in the extracellular domain, at the interface of two adjacent subunits [1]. However, in addition to this well-characterised orthosteric binding site, nAChRs can be modulated by the binding of ligands to distinct allosteric binding sites [2], [3]. Indeed, modulation by allosteric ligands appears to be a feature that is characteristic of ligand-gated ion channels [4]. In recent years, a diverse array of nicotinic allosteric modulators have been described [2], [5], some of which have been reported to interact with an intrasubunit transmembrane binding site [6]–[8].

A notable feature of α7 nAChRs is the rapid rate of receptor desensitisation induced by conventional orthosteric agonists [9], a phenomenon that is also influenced by temperature [10]. As is now well established, the extent of α7 nAChR desensitisation can also be influenced by allosteric modulators. Two types of positive allosteric modulators (PAMs) of α7 nAChRs have been described: those with minimal effects on receptor desensitisation (‘type I’ PAMs) and those that greatly reduce or abolish agonist-induced desensitisation (‘type II’ PAMs) [2]. There is evidence that compounds classified as being either type I or type II PAMs bind competitively at a common or overlapping allosteric site on α7 nAChRs [11] and do so at a site in the receptor’s transmembrane region [6], [11]. It also seems likely that this classification is an over-simplification because PAMs acting on α7 nAChRs with a wide-spectrum of effects on receptor desensitisation have been reported [8]. Additionally, there is evidence that ligands binding at this allosteric transmembrane site on α7 nAChRs can result in receptor activation in the absence of a conventional orthosteric agonist and can cause activation that is associated with minimal levels of receptor desensitisation [7], [8]. Whereas allosteric modulators that lack agonist activity have been described as PAMs, the term allosteric agonist has been used to describe ligands that bind to an allosteric site but, unlike PAMs, have agonist activity in the absence of an orthosteric agonist [7].

A major goal, both for academic research and for pharmaceutical drug discovery is the identification and characterisation of ligands that are selective for particular receptor subtypes. In the case of α7 nicotinic acetylcholine receptors (nAChRs), interest in subtype-selective agonists and allosteric modulators has arisen, at least in part, as a consequence of these receptors having been implicated in a range of neurological and psychiatric disorders [5], [12], [13]. Here we describe studies conducted with three pharmacologically distinct α7-selective nicotinic ligands: an orthosteric agonist, a positive allosteric modulator and a non-desensitising allosteric agonist. The main motivation for the experiments described in the present study is to examine the suitability of these three pharmacologically distinct types of ligand for the characterisation of native nAChRs. In addition we have undertaken studies with recombinant nAChRs to assess the subtype-selectivity of these compounds.

A large number of α7-selective nAChR agonists have been identified in recent years [14], [15], but one that has been studied extensively is the biarylcarboxamide compound (R)-N-(1-azabicyclo[2.2.2]oct-3-yl)(5-(2-pyridyl)thiophene-2-carboxamide) [16]. It has been described in the scientific literature, somewhat inconsistently, as either ‘compound A’ [17]–[20] or as ‘compound B’ [21]–[24]. Here, we used the nomenclature ‘compound B’, the name that was assigned first to this molecule in the scientific literature.

In addition to studies with the orthosteric agonist compound B, we have also examined the pharmacological properties of two allosteric modulators of α7 nAChRs: 4-(1-napthyl)-3*a*,4,5,9*b*-tetrahydro-3*H*-cyclopenta[*c*]quinoline-8-sulfonamide (TQS) [7], [25] and 4-(4-bromophenyl)-3*a*,4,5,9*b*-tetrahydro-3*H*-cyclopenta[*c*]quinoline-8-sulfonamide (4BP-TQS) [7], [8]. TQS has no agonist activity on α7 nAChRs when applied alone but, as has been reported previously, it is a potent PAM of α7 nAChRs [7], [25]. In addition to potentiating agonist-evoked responses when co-applied with an orthosteric agonist, co-application of TQS also greatly reduces the rate of desensitisation of α7 nAChRs [7], [25]. As explained above, this has resulted in it being described as a ‘type II’ PAM [2], [25]. 4BP-TQS has close chemical similarity to TQS but acts as a potent allosteric agonist of α7 nAChRs, as has been shown previously in studies conducted with recombinant receptors expressed *Xenopus laevis* oocytes [7], [8]. In contrast to orthosteric agonists, activation of α7 nAChRs by 4BP-TQS is associated with minimal levels of receptor desensitisation [7], [8].

In this paper we have characterised, utilising recombinant nAChRs receptors, the selectivity of compound B, TQS and 4BP-TQS for α7 nAChRs. In addition, we describe studies examining the suitability of these compounds for the pharmacological characterisation of native nAChRs expressed in rat primary hippocampal cell cultures and also in human iCell neurons.

## Results

### Subtype Selectivity of Compound B, TQS and 4BP-TQS

**Figure 1:**
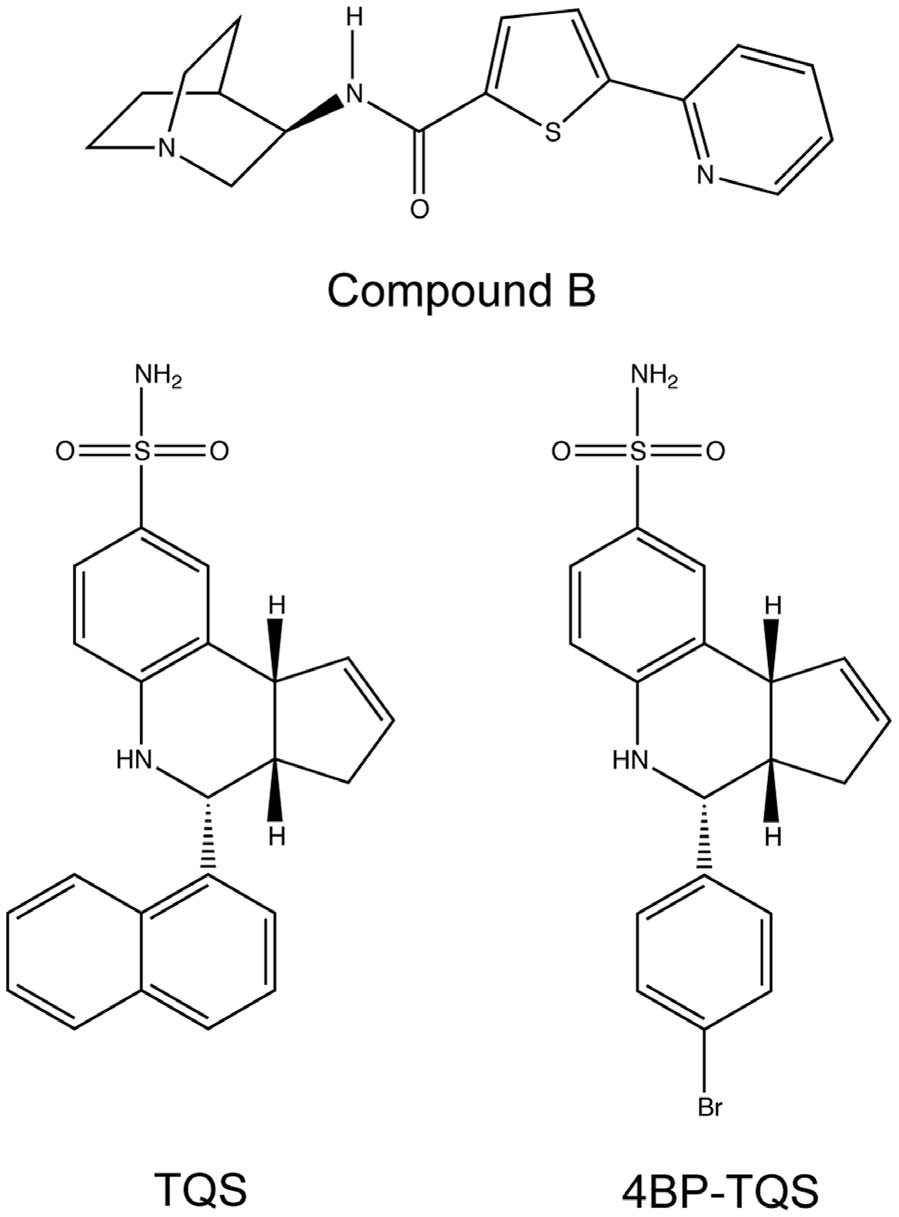
Chemical structure of α7 nAChR orthosteric and allosteric ligands examined in the present study.

**Figure 2:**
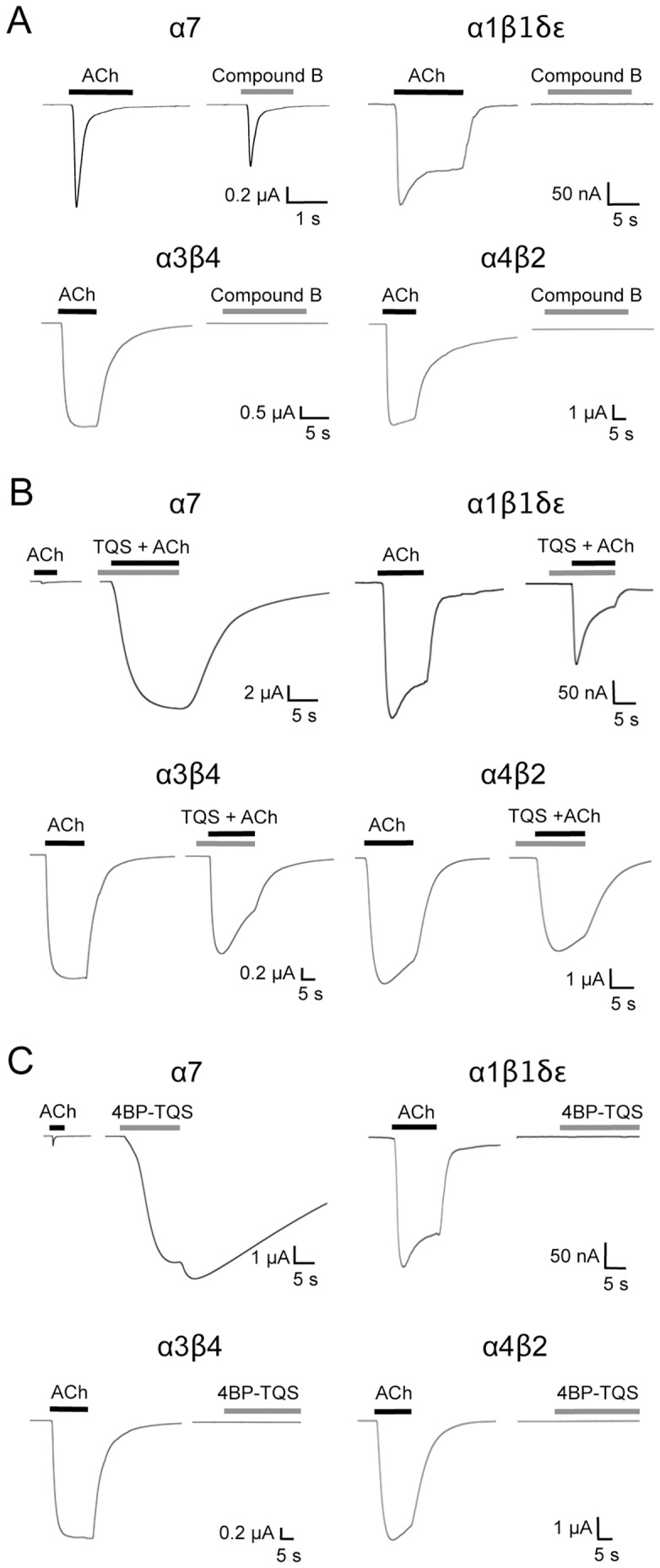
Subtype selectivity of compound B, TQS and 4BP-TQS examined with human recombinant nAChR subtypes expressed in *Xenopus* oocytes. A) Responses to ACh (3 mM on α7 nAChRs and 100 µM on other nAChR subtypes; black bars) and to compound B (30 µM; grey bars). B) Responses to ACh (100 µM) and to ACh (100 µM; black bars) pre- and co-applied with TQS (100 µM; grey bars). C) Responses to ACh (3 mM on α7 nAChRs and 100 µM on other nAChR subtypes; black bars) and to 4BP-TQS (60 µM on α7 nAChRs and 100 µM on other nAChR subtypes; grey bars).

In previous studies the orthosteric agonist compound B (Figure 1) has been described as being selective for α7 nAChRs [17]–[19], [21]. However, there is only a very limited amount of published experimental data demonstrating that compound B lacks agonist activity on nAChR subtypes other than α7. Here we have examined the ability of compound B to activate several human recombinant nAChR subtypes expressed in *Xenopus* oocytes. Data obtained from these studies supports the conclusion that compound B is an α7-selective agonist (Figure 2A). As is seen with ACh, activation of human α7 nAChRs by compound B results in rapid activation associated with fast desensitisation (Figure 2A). However, compound B acted as a partial agonist: maximal concentrations of compound B (30 µM) generating responses that were 61±10% (n = 6) of the responses with a maximal concentration of ACh. In contrast, compound B had no agonist activity on muscle (α1β1δε) or neuronal (α3β4 or α4β2) nAChRs (Figure 2A; Table 1).

**Table 1:** Pharmacological properties of TQS and 4BP-TQS on nAChR subtypes.

Receptor subtype	TQS		4BP-TQS	
	Fold potentiation*	% Inhibition**	Agonist response†	% Inhibition††
α1β1δε	−	27±2 (*n* = 5)	−	24±5 (*n* = 5)
α3β4	−	37±3 (*n* = 4)	−	36±10 (*n* = 4)
α4β2	−	9±3 (*n* = 3)	−	9±4 (*n* = 3)
α7	28±6 (*n* = 13)	−	38±6 (*n* = 23)	−

* Fold potentiation of response to ACh (100 µM) by TQS (100 µM).

** Percentage inhibition of response to ACh (100 µM) by TQS (100 µM).

† Agonist response of 4BP-TQS (100 µM) expressed as a fold response normalised to maximal concentration of ACh (3 mM).

†† Percentage inhibition of response to ACh (100 µM) by 4BP-TQS (100 µM). Data are means ± SEM.

TQS (Figure 1) has been shown previously to be a selective positive allosteric modulator (PAM) of α7 nAChRs [25]. In contrast, to its potentiating effects on α7 nAChRs, TQS has been reported to cause inhibition of agonist-evoked responses with α3β4 and α4β2 nAChRs [25]. Our studies, conducted with human recombinant α3β4 and α4β2 nAChRs expressed in *Xenopus* oocytes (Figure 2B; Table 1), agree with this previously published data, with particularly strong inhibition being observed with α3β4 nAChRs (Table 1). In addition, an inhibitory effect of TQS was also observed on α1β1δε muscle nAChRs (Figure 2B; Table 1).

More recently, it has been demonstrated that 4BP-TQS (Figure 1), a compound with close chemical similarity to TQS, is an allosteric agonist of α7 nAChRs [7], [8]. In contrast to the rapid rate of desensitisation observed when α7 nAChRs are activated by orthosteric agonists such as ACh or compound B, little or no desensitisation is observed in response to activation by 4BP-TQS (Figure 2C). However, in previous studies, the selectivity of 4BP-TQS for nAChR subtypes has not been examined. Here, we have examined the ability of 4BP-TQS to activate a range of human recombinant nAChRs expressed in *Xenopus* oocytes (Figure 2C). 4BP-TQS had no agonist activity on muscle (α1β1δε) or neuronal (α3β4 or α4β2) nAChRs (Figure 2C). In addition, as was observed with TQS, co-application of 4BP-TQS caused inhibition of responses to ACh on α3β4, α4β2 and α1β1δε nAChRs (Table 1).

In summary, these data support the conclusion that compound B, TQS and 4BP-TQS are selective agonists or potentiators of α7 nAChRs.

### Characterisation of α7-selective Compounds on Native α7 nAChRs by Patch-clamp Recording

**Figure 3:**
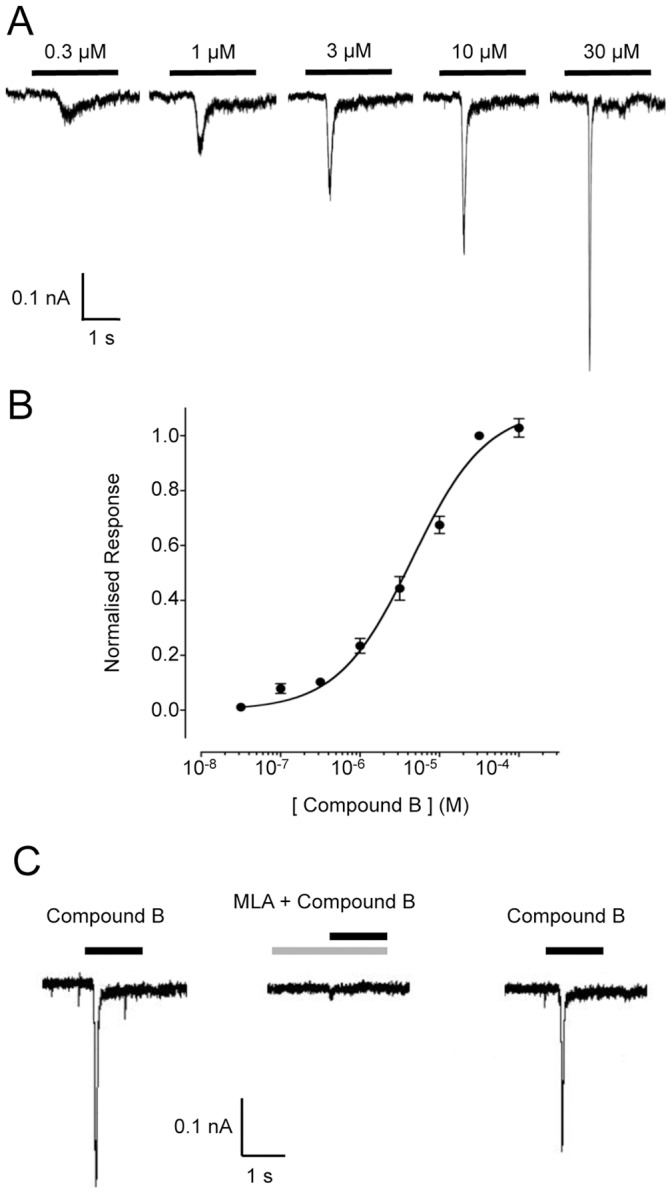
Agonist activation of α7 nAChRs by compound B examined by patch-clamp in rat primary hippocampal cells. A) Representative recordings showing responses to the application of compound B (0.3 to 30 µM; black bars). B) Dose-response data are presented for compound B (0.03 to 100 µM). Data are normalised to 30 µM compound B. Data are means ± SEM of 5 independent experiments. C) Responses to compound B (10 µM, black bars) were blocked by the α7-selective antagonist methyllycaconitine (MLA; 10 nM, grey bar).

The ability of compound B to activate native α7 nAChRs in rat primary hippocampal neurons was examined by patch-clamp recording (Figure 3). Rapidly desensitising dose-dependent responses, characteristic of α7 nAChRs, were observed (Figure 3A). Compound B activated rat hippocampal nAChRs with an *EC*_50_ value of 4.5±1.1 µM (n = 4) and a Hill coefficient of 0.9±0.05 (n = 4) (Figure 3B). Responses to compound B in rat primary hippocampal neurons were blocked by the α7-selective nAChR antagonist methyllycaconitine (MLA) (Figure 3C).

**Figure 4:**
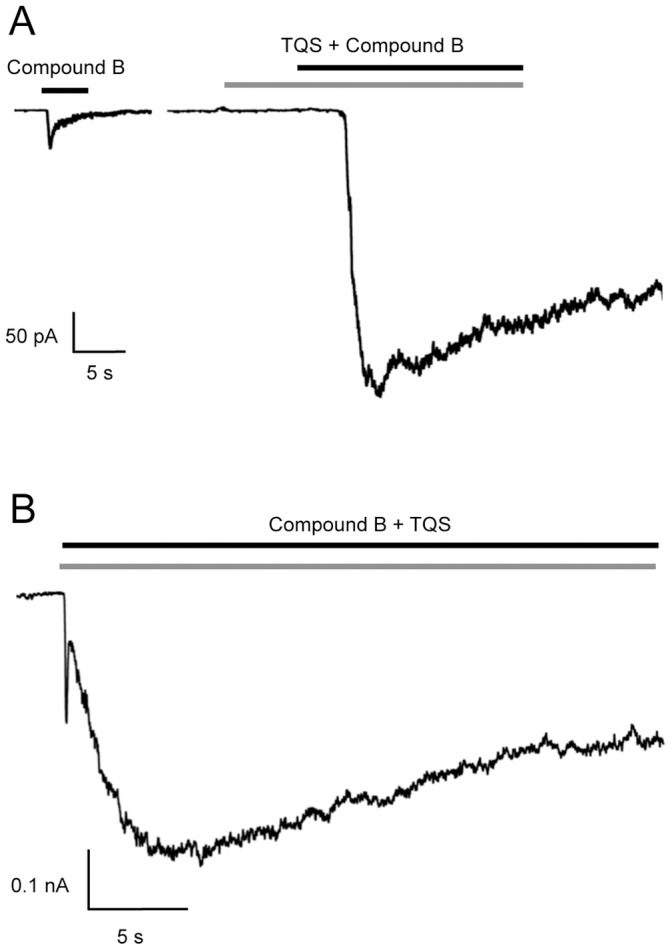
Positive allosteric modulation of α7 nAChRs by TQS examined by patch-clamp in rat primary hippocampal cells. A) Representative recordings showing responses to the application of compound B (1 µM; Left; black bar) and of TQS (10 µM; grey bar) pre-applied for 5 s and then co-applied with compound B (1 µM; Right; black bar). B) Co-application of compound B (1 µM; black bar) with TQS (10 µM; grey bar) results in a two-component response.

Previous studies have shown that TQS is a strong potentiator of recombinant α7 nAChRs, causing a dramatic reduction in orthosteric agonist-induced receptor desensitisation [7], [8], [25]. The ability of TQS to potentiate compound B-evoked currents was examined in rat primary hippocampal neurons (Figure 4). When a maximal concentration of TQS (10 µM) was pre-applied and then co-applied with an EC_20_ concentration of compound B, responses were potentiated by 5.2±1.8 fold (n = 6) (Figure 4A). When TQS was co-applied with compound B, without a pre-application a two-component response was observed (Figure 4B). As has been suggested previously from studies with recombinant α7 nAChRs, it is likely that this two-component response is due to a rapid interaction of the orthosteric agonist with its extracellular binding site, followed by a slower interaction of TQS with its transmembrane allosteric binding site [7].

**Figure 5:**
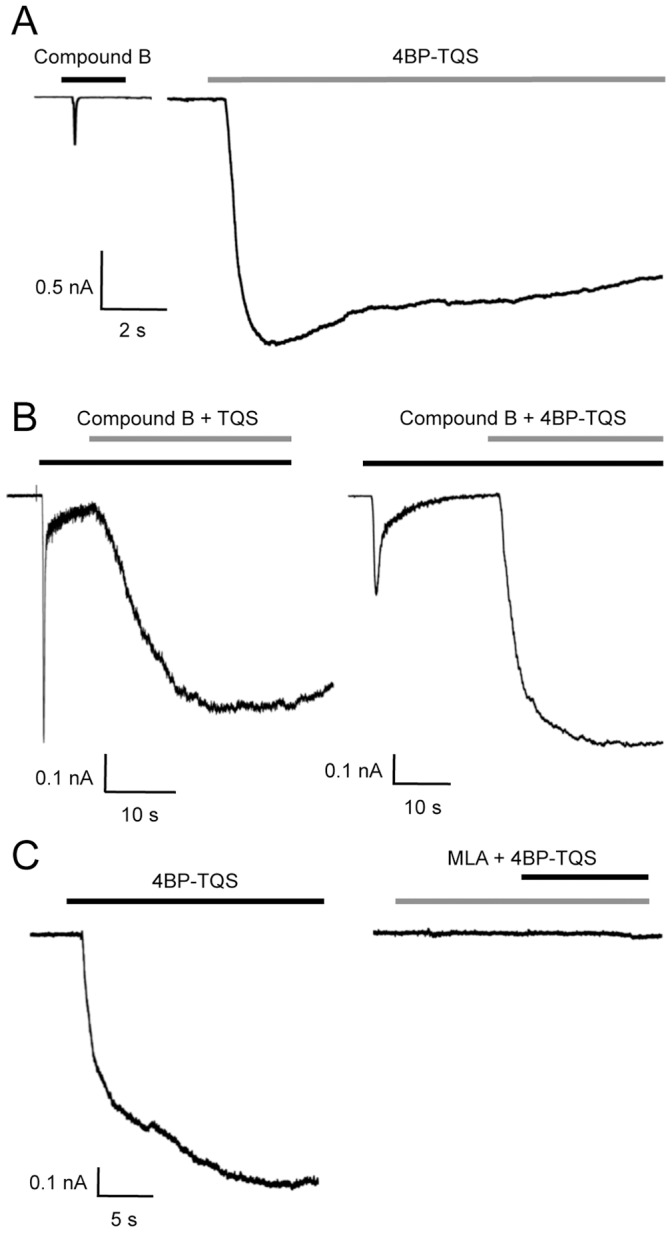
Characterisation of 4BP-TQS on α7 nAChRs examined by patch-clamp recording of rat primary hippocampal cells. A) Representative recordings showing responses to the application of compound B (30 µM; Left; black bar) and of 4BP-TQS (30 µM; Right; grey bar). B) Prolonged exposure of α7 nAChRs to compound B (1 µM; black bar) results in receptor activation, followed by rapid desensitisation. In continued presence of compound B (1 µM; black bar), co-application of either TQS (1 µM; Left; grey bar) or 4BP-TQS (30 µM; Right; black bar) resulted in reactivation of desensitised receptors. C) Responses to 4BP-TQS (30 µM; Left; black bar) are blocked by MLA (10 nM; grey bar) when MLA was pre-applied for 15 s and then co-applied with 4BP-TQS (30 µM; black bar).

Previous studies with recombinant nAChRs have demonstrated that 4BP-TQS is a potent allosteric agonist that activates α7 nAChRs with minimal desensitisation [7], [8]. We have now examined native nAChRs expressed in rat hippocampal neurons and observed a similarly dramatic difference in responses evoked with the orthosteric agonist compound B and the allosteric agonist 4BP-TQS (Figure 5). Peak responses to a maximal concentration of 4BP-TQS (30 µM) were 3.5±0.6 (n = 9) fold larger than responses to a maximal concentration of compound B (30 µM). In addition, as seen previously with recombinant α7 nAChRs [7], [8] responses to 4BP-TQS had a slower onset and resulted in a very much slower rate of desensitisation than is observed with orthosteric agonists (Figure 5A).

Another feature of both TQS and 4BP-TQS that has been observed with recombinant nAChRs is their ability to facilitate recovery of α7 nAChRs from desensitisation induced by orthosteric agonists [7], [8]. We here confirm that the same effect can be seen by co-applying either TQS or 4BP-TQS to native hippocampal nAChRs after desensitisation with compound B (Figure 5B). After responses had fully desensitised in the continued presence of compound B, recovery from desensitisation was observed with both allosteric modulators (Figure 5B). Also, as expected from previous studies with recombinant α7 nAChRs, responses to 4BP-TQS were completely blocked by the α7-selective antagonist MLA (Figure 5C).

### Characterisation α7-selective Compounds on Native α7 nAChRs by Calcium Imaging

**Figure 6:**
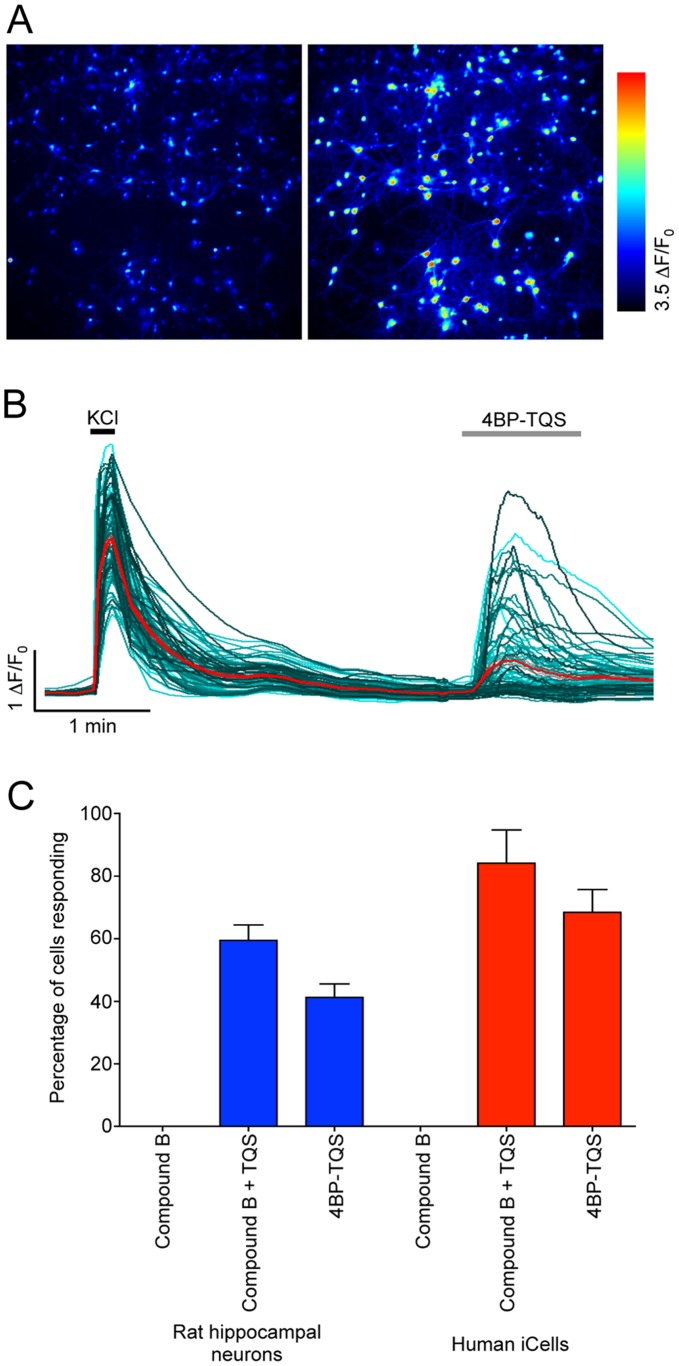
Characterisation of native nAChRs in rat primary hippocampal cells and human induced pluripotent stem cell-derived neurons, examined by fluorescence-based intracellular calcium imaging. A) Pseudocolour images of rat hippocampal neurons corresponding to low initial resting calcium levels (Left) and higher calcium levels, after application of 30 µM 4BP-TQS (Right). B) Single cell traces (cyan) for all neurons present in the optical field. The average response is shown in red (n = 89 cells). C) Histogram illustrating the percentage of cells that responded to compound B (1 µM), compound B co-applied with TQS (1 µM and 10 µM, respectively) and 4BP-TQS (30 µM) in rat primary hippocampal cells (blue) and in iCell neurons (red). Data were normalised to the total number of cells that responded to KCl (50 mM) (n = 3–31).

**Figure 7:**
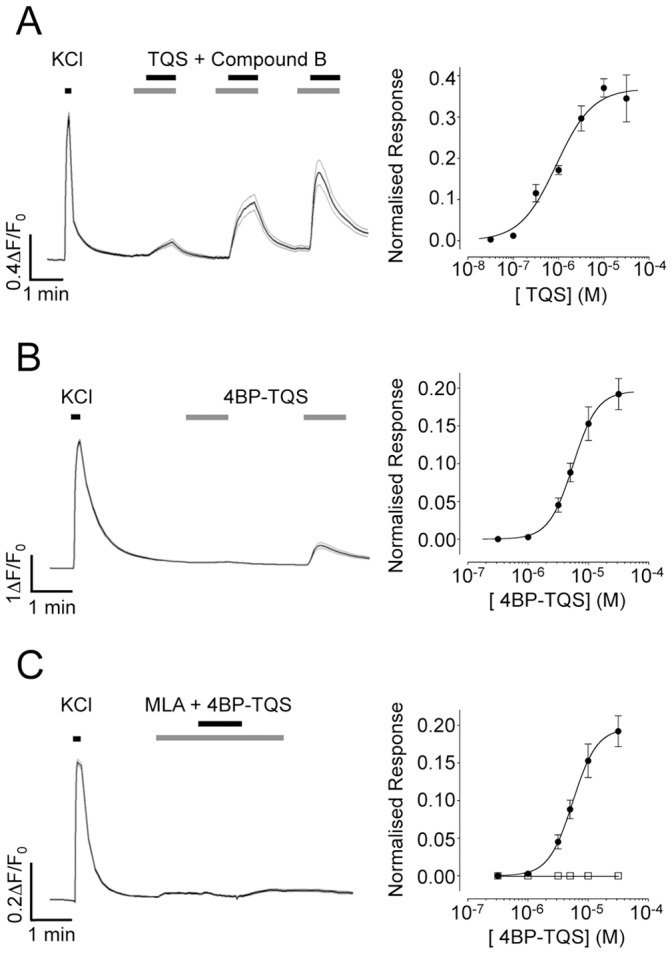
Characterisation of TQS and 4BP-TQS on α7 nAChRs in rat primary hippocampal cells, examined by fluorescence-based intracellular calcium imaging. A) Potentiation by TQS of responses evoked by compound B. TQS was pre-applied and then co-applied (0.3 µM, 3 µM, and 30 µM) with compound B (5 nM; Left). The trace represents the average response of 66 individual traces ± SEM. Dose-response data are presented (Right) for a range of concentrations of TQS (0.03 to 30 µM) in the presence of compound B (5 nM). B) Calcium-responses in response to application of 4BP-TQS (1 and 30 µM) (Left). The trace represents the average response of 89 individual traces ± SEM. Dose response data are presented (Right) for a range of concentrations of 4BP-TQS (0.3 to 30 µM). C) Responses to 4BP-TQS (30 µM) were blocked by MLA (100 nM) when MLA was pre-applied and then co-applied with 4BP-TQS (30 µM; Left). The trace represents the average response of 106 individual traces ± SEM. Dose response data are presented for a range of concentrations of 4BP-TQS in the absence (circles) or presence (squares) of MLA (0.7 nM). In all cases, MLA was pre-applied for 30 s and then co-applied with 4BP-TQS. All responses were normalised to KCl (50 mM). Dose-response data are means ± SEM of 3–7 independent experiments.

The suitability of compound B, TQS and 4BP-TQS as tools for the pharmacological characterisation of endogenous nAChRs expressed in primary rat hippocampal neurons was also examined by fluorescence-based intracellular calcium imaging. We did not detect an agonist-induced intracellular calcium response in primary rat hippocampal neurons with compound B (Figure 6), presumably because of the low open probability and rapid desensitisation of α7 nAChRs in response to activation with orthosteric agonists compared to that observed in the presence of type II PAMs or allosteric agonists [26], [27]. Similarly, and as expected by a pure potentiator, no response was detected when TQS was applied alone. In contrast, a strong dose-dependent increase in intracellular calcium was observed when compound B (5 nM) was co-applied with TQS (Figure 7A). Responses to the co-application of compound B and TQS were observed in 59±5% of cells (n = 10) (Figure 6B) and experiments conducted with a range of TQS concentrations revealed an EC_50_ value of 0.9±0.3 µM (n = 3) (Figure 7A).

In contrast to the lack of effects on intracellular calcium responses in primary hippocampal neurons with the orthosteric agonist compound B, clear agonist-evoked responses were detected with 4BP-TQS (Figure 6 and 7B) in 41±4% of cells examined (n = 31). 4BP-TQS activated native nAChRs with an EC_50_ value of 5.5±1.8 µM and a Hill coefficient of 2.2±0.6 (n = 6; Figure 7B). In addition, responses to 4BP-TQS were blocked completely by the α7-selective antagonist MLA (Figure 7C). Antagonism by MLA was dose-dependent, with an IC_50_ value of 0.76±0.09 nM (Data not shown). Studies conducted with a range of concentrations of 4BP-TQS demonstrated that antagonism by MLA was not surmountable (Figure 7C), consistent with previous evidence that MLA and 4BP-TQS do not bind competitively at a common site [7].

**Figure 8:**
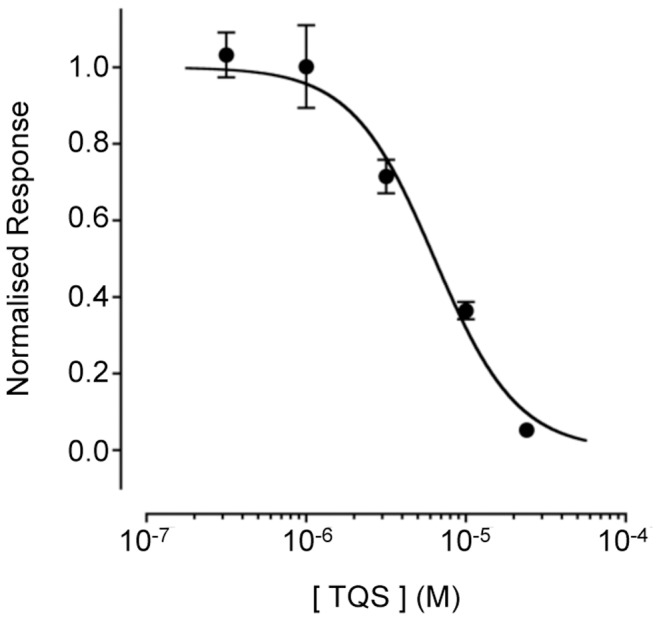
Block of 4BP-TQS responses by TQS in rat primary hippocampal cells examined by fluorescence-based intracellular calcium imaging. Dose response data are presented for a range of concentrations of TQS (0.3 to 30 µM) in the presence of 4BP-TQS (10 µM). In all cases TQS was pre-applied for 30 s and then co-applied with 4BP-TQS. Responses were normalised to 4BP-TQS (10 µM). Data are means ± SEM of 3 independent experiments.

Responses evoked by 4BP-TQS (10 µM) were also blocked in a dose-dependent manner by TQS with an IC_50_ value of 6.1±0.4 µM (n = 3; Figure 8). This is consistent with previous studies indicating that TQS and 4BP-TQS bind competitively at a common or overlapping allosteric site [7], [8].

### Functional Characterisation of Native nAChRs in Human iCell Neurons

**Figure 9:**
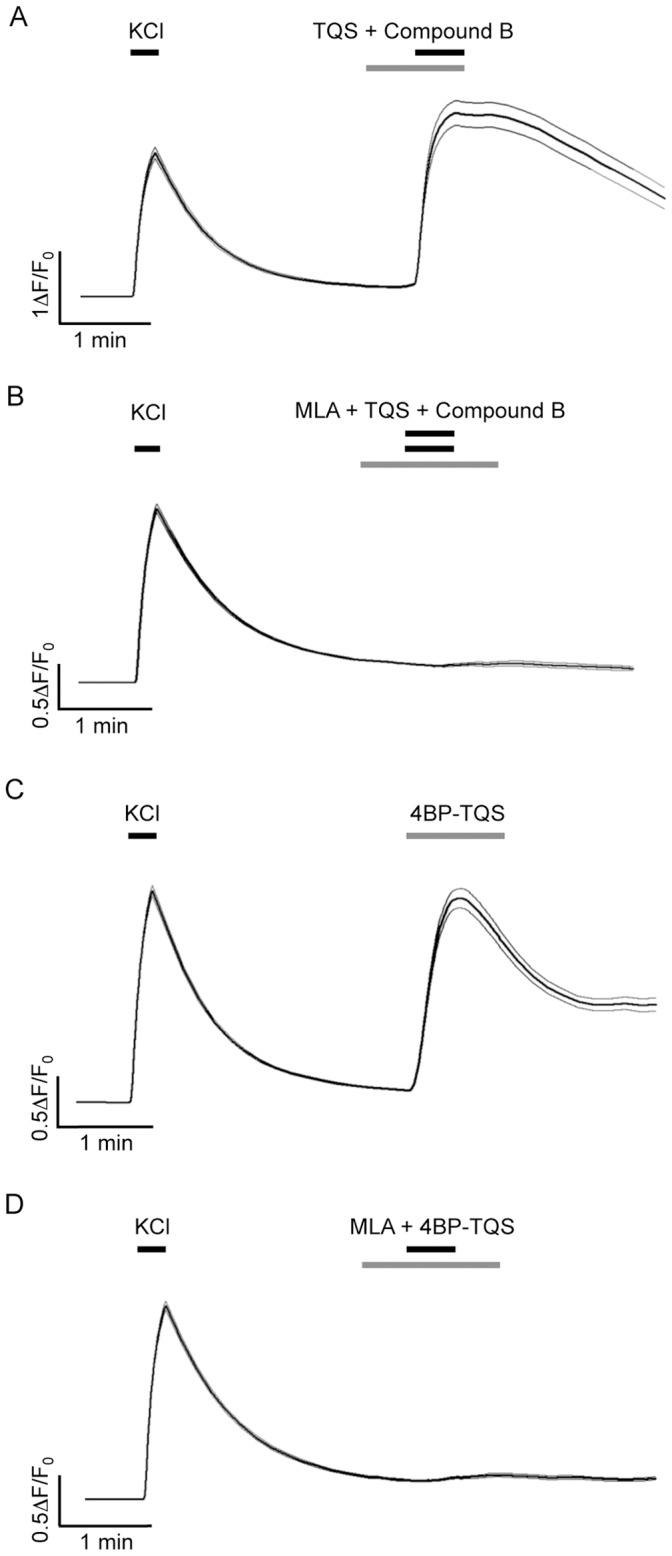
Characterisation of TQS and 4BP-TQS in human induced pluripotent stem cell-derived neurons examined by fluorescence-based intracellular calcium imaging. A) Pre- and co-application of TQS (30 µM) with compound B (30 nM) potentiates the amplitude of compound B -evoked calcium-responses (almost undetectable in the absence of TQS) (Left). The trace represents the average response of 35 individual traces ± SEM. Responses to compound B (30 µM) co-applied with TQS (30 µM) are blocked when pre- and co-applied with MLA (1 µM; Right). The trace represents the average response of 137 individual traces ± SEM. B) Agonist-evoked calcium-responses were detectable in response to the allosteric agonist 4BP-TQS (30 µM; Left). The trace represents the average response of 122 individual traces ± SEM. Responses to 4BP-TQS (30 µM) were blocked by pre- and co-application of MLA (1 µM; Right). The trace represents the average response of 118 individual traces 5± SEM.

The successful demonstration of functional nAChRs in primary rat hippocampal neurons by calcium imaging, prompted us to examine whether this technique could be used to determine whether functional nAChRs are present in human induced pluripotent stem cell-derived (iCell) neurons. Agonist-evoked elevations in intracellular calcium were observed in response to 4BP-TQS and also when compound B was co-applied with TQS (Figure 9). Responses to the co-application of compound B and TQS were observed in 84±11% of cells (n = 3) and to 4BP-TQS in 68±7% of cells (n = 3) (Figure 6C). In both cases, agonist-evoked responses were blocked by MLA (Figure 9). Thus, the combined use of iCell neurons and α7-selective allosteric modulators provides a convenient approach to the rapid fluorescence-based characterization of native human α7 nAChRs.

## Discussion

Nicotinic receptors are important targets for pharmaceutical drug discovery [14], [15]. For example, nAChRs expressed at the neuromuscular junction (α1β1δε) are targets for muscle relaxant drugs and nAChR subtypes in the brain (such as α4β2) are targets for drugs used to aid smoking cessation. In addition, neuronal nAChR subtypes such as α7 have been identified promising targets for drug targets in a variety of neurological and psychiatric disorders [12], [28]. As a consequence, considerable efforts have been focussed on the identification of compounds that are selective for α7 nAChRs [5]. These include conventional orthosteric agonists as well as allosteric modulators of α7 nAChRs that either potentiate orthosteric agonists or are able to activate α7 nAChRs via an allosteric site [5], [12], [28].

It is well-established that orthosteric agonists such as ACh bind to an extracellular site at the interface between two subunits [29]. In contrast, there is evidence that PAMs such as TQS and allosteric agonists such as 4BP-TQS bind at an intra-subunit site in the nAChR transmembrane domain [6]–[8]. In addition to being allosteric modulators of orthosteric agonists, PAMs such as TQS block responses to allosteric agonists [8]. Experimental data and computer docking simulations are consistent with these two classes of allosteric modulator (PAMs and allosteric agonists) binding at a common site [6], [7] but it is possible that such effects could result from TQS and 4BP-TQS binding to two distinct non-overlapping allosteric sites.

Because of the extensive subunit diversity of nAChRs [30], it is important to establish the subtype selectivity of such ligands. In this study we have focussed on three previously described α7 nAChR ligands, an orthosteric agonist (compound B), a positive allosteric modulator (TQS) and an allosteric agonist (4BP-TQS). Studies conducted with recombinant nAChRs expressed in *Xenopus* oocytes have enabled us to demonstrate that all three of these ligands show a high degree of selectivity for α7 nAChRs (Figure 2). The availability of subtype-selective ligands is particularly important for the characterisation of native nAChRs, due to the likelihood that neurons may express a heterogeneous mixture of receptor subtypes of uncertain subunit composition [30], [31]. In the present study we have exploited the availability of α7-selective ligands to characterise native α7 nAChRs. Initial studies, conducted by patch-clamp electrophysiology demonstrated that all three α7-selective ligands (compound B, TQS and 4BP-TQS) exhibited properties on native α7 nAChRs similar to those reported previously on recombinant receptors. In addition, these studies have been extended to examine the suitability of these compounds for fluorescence-based techniques. In part, the rationale for these studies is the usefulness of fluorescence-based assays for high-throughput studies aimed at receptor identification and characterisation.

Patch-clamp studies conducted with rat hippocampal neuronal neurons have confirmed the ability of TQS to potentiate native α7 nAChRs and to reduce the rapid rate of desensitisation induced by orthosteric agonists (Figure 4A). In addition, for the first time, we have demonstrated the ability of an allosteric agonist (4BP-TQS) to activate native α7 nAChRs. As reported previously in studies with recombinant α7 nAChRs [7], 4BP-TQS activated native nAChRs in rat hippocampal cells without inducing the rapid agonist-induced desensitisation seen with orthosteric agonists (Figure 5A). Data obtained with native α7 nAChRs has also confirmed the ability of both TQS and 4BP-TQS to reactivate native α7 nAChRs after orthosteric agonist-induced desensitisation (Figure 5B). All of these findings are consistent with previous studies conducted with recombinant α7 nAChRs [7], [8], [25].

Due to the low open probability and rapid rate of desensitisation of α7 nAChRs, conventional orthosteric agonists are not well suited to fluorescence-based functional assays. However, previous studies with recombinant α7 have demonstrated that α7 responses can be detected in fluorescence-based assays when conventional agonists are co-applied with potentiators that reduce rates of receptor desensitisation [32]–[35]. Here we have demonstrated similar effects with two cell types expressing native α7 nAChRs, rat hippocampal neuronal cells and human induced pluripotent stem cell-derived neurons. The identification of tools and techniques suitable for detection, in fluorescence-based assays, of α7 nAChRs expressed endogenously in cell preparations such as primary hippocampal neurons and iCell neurons will be of considerable assistance for both academic research and pharmaceutical drug discovery.

Although there have been previous studies that have examined the properties of α7 nAChRs PAMs on native nAChRs [32], [34], [36], [37], we are not aware of any such studies with α7 nAChR allosteric agonists. Our patch-clamp electrophysiological studies reveal (Figures 4 and 5) that both TQS and 4BP-TQS behave in a similar manner on native α7 nAChRs as they do on recombinant α7 nAChRs [7], [8], [25]. Significantly, we have also demonstrated that non-desensitising allosteric agonists, such as 4BP-TQS, enable α7 nAChRs expression and function to be examined by fluorescence-based assays without the need for a second potentiating ligand (Figure 6 and 7).

Historically, the very rapid desensitisation of α7 nAChRs caused some controversy regarding the properties of these receptors. For example, prior to the molecular cloning of α7, there were known to be binding sites for the snake neurotoxin α-bungarotoxin in both the mammalian brain and at the neuromuscular junction. However, functional nAChRs, blocked by α-bungarotoxin, had not been detected in the brain. This led to the suggestion that brain α-bungarotoxin binding sites did not correspond to functional nAChRs [27], an idea that persisted until the cloning and functional characterization of recombinant α7 nAChRs [9]. The availability of ligands that activate α7 without inducing rapid desensitisation has considerable potential benefits for the identification and characterisation of endogenous α7 nAChRs in native cell preparations.

This study has confirmed the subtype selectivity of three nAChR ligands that exhibit distinct pharmacological properties. In addition, whereas previous studies reporting the effects of allosteric agonists such as 4BP-TQS on α7 nAChRs have focussed exclusively on recombinant receptors [7], [8], [10], [27], here we have confirmed that allosteric agonists display similar pharmacological properties on native nAChRs to those reported previously on recombinant receptors. We have also demonstrated that, whereas orthosteric agonists of α7 nAChRs are not well-suited to fluorescence-based assays, clear fluorescence responses can be detected either by the use of an allosteric agonist (4BP-TQS) or by the co-application of a type II PAM (TQS). Finally, by the combined use of a non-desensitising subtype-selective allosteric modulators and fluorescence-based calcium imaging techniques, we have been able to demonstrate, for the first time, the expression of α7 nAChRs in human induced pluripotent stem cell-derived neurons. This is important, given the need in pharmaceutical drug discovery to identify convenient sources of human native neuronal nAChRs for compound screening and testing.

## Materials and Methods

### Chemical Synthesis

(R)-N-(1-azabicyclo[2.2.2]oct-3-yl)(5-(2-pyridyl)thiophene-2-carboxamide) (compound B) was synthesised by Lilly Research Laboratories according to methods described previously [16]. 4-(1-napthyl)-3*a*,4,5,9*b*-tetrahydro-3*H*-cyclopenta[*c*]quinoline-8-sulfonamide (TQS) and 4-(4-bromophenyl)-3*a*,4,5,9*b*-tetrahydro-3*H*-cyclopenta[*c*]quinoline-8-sulfonamide (4BP-TQS) were obtained from Chembridge Corporation (San Diego, CA).

### Subunit cDNAs and Plasmid Expression Vectors

Human α3, α4, β2 and β4 nAChR subunit cDNAs [38] in the plasmid pcDNA3.1 were obtained from Merck Research Laboratories (La Jolla, CA). Human α7 nAChR subunit cDNA in the plasmid pSP64GL has been described previously [7], [39]. Human α1, β1, δ and ε subunit cDNAs [40] in the plasmid pcDNA3.1 were kindly provided by David Beeson, University of Oxford.

### *Xenopus* Oocyte Electrophysiology

Adult female *Xenopus laevis* frogs were obtained from the European *Xenopus* Resource Centre (University of Portsmouth). *Xenopus laevis* oocytes were isolated and defolliculated as described previously [41] following procedures that have been approved by both UCL’s Biological Services Management Group and the UK Home Office (under licences PIL70/23585 and PPL70/06819). To express human α7 nAChRs, *in vitro* transcribed cRNA was injected into the oocyte cytoplasm. *In vitro* transcription of cRNA was carried out using the mMESSAGE mMACHINE SP6 transcription kit (Ambion, Huntington, UK). Oocytes were injected with 6–12 ng cRNA per oocyte in a volume of 32.2 nl using a Drummond variable volume microinjector. To express human α4β2 and α3β4 nAChRs, plasmid cDNA constructs were co-injected into the oocyte nucleus in a 1∶1 ratio, and approximately 10 ng of cDNA was injected in a total injection volume of 32.2 nl per oocyte. Two electrode voltage-clamp recordings were performed (with the oocyte membrane potential held at −60 mV), as described previously [41] using a Warner Instruments OC-725C amplifier (Harvard Apparatus, Edenbridge, UK), PowerLab 8SP and Chart 5 software (AD Instruments, Oxford, UK). Agonists and allosteric modulators were applied to oocytes using a BPS-8 solenoid valve solution exchange system (ALA Scientific Inc., Westbury, NY), controlled manually or by Chart software.

### Cell Culture

Hippocampal tissue, isolated from E18 Sprague Dawley rat brain, was purchased from Charles River Laboratories (Margate, UK). Typically, 8–10 hippocampi were dissociated in 10 ml trypsin-EDTA for 10 min at 37°C. The trypsin-EDTA solution was replaced with 5 ml neurobasal medium (Invitrogen, Paisley, UK) supplemented with 10% heat-inactivated foetal bovine serum (FBS, PAA Laboratories GmbH), B-27 supplement (Invitrogen, Paisley, UK) and 29.2 mg/ml L-glutamine (PAA Laboratories GmbH). The neurobasal medium was then replaced with 2.5 ml Hank's balanced salt solution (HBSS, Invitrogen, Paisley, UK) supplemented with 20 mg/ml DNase (Sigma-Aldrich, Poole, UK) and gently triturated by suction using a 1 ml Gilson pipette. Once the cells were dissociated, 7.5 ml HBSS with DNase was added and centrifuged for 5 min at 5°C and 200×*g* (RCF). For patch-clamp experiments, dissociated cells were plated at a density of approximately 1×10^5^ cells/ml (100 µl/cover slip) on Biocoat poly-D-lysine (PDL)/laminin coverslips (Becton Dickinson Biosciences, Oxford, UK). For Ca^2+^ imaging experiments, dissociated cells were plated at a density of approximately 3×10^4^ cells/ml into Biocoat PDL 96-well plates (100 µl/well).

Human neuronal cells, derived from induced pluripotent stem cells (iCell neurons), were obtained from Cellular Dynamics International (Madison, WI). Cells were plated at 3×10^4^ cells/ml in Biocoat PDL 96-well plates (10 µl/well) and maintained at 37°C (5% CO_2_) in serum-free Neurobasal-B27 medium, as above. Calcium imaging experiments were performed on these cells after 6–9 days in culture.

### Patch-clamp Recording

Whole-cell voltage-clamp recordings were carried out 6–10 days after plating. Cells selected for patch-clamp recordings had somatic diameters of 15–30 µM, with neurite extensions. During recordings, cells were continuously perfused in HEPES-buffered Tyrode’s solution (HBTS, Invitrogen, Paisley, UK) containing (mM): 135 NaCl, 5 KCl, 1.2 MgCl_2_, 2.5 CaCl_2_, 10 HEPES, 11 glucose, 0.001 tetrodotoxin (with citrate) (TTX), pH = 7.2 at room temperature. Cells were voltage-clamped in the whole-cell configuration (at holding potential of -60 mV) with an AxoPatch 200A patch-clamp amplifier (Molecular Devices, Sunnyvale, CA, USA). Pipettes were pulled from borosilicate glass (Type GC150F-10, Harvard Apparatus, Kent, UK) using a commercial puller (Model P-87, Sutter Instruments, Novato, CA, USA) and had resistances between 2 and 6 MΩ when filled with pipette solution containing (mM): 1 MgCl_2_, 4 MgATP, 0.5 EGTA, 10 HEPES, 140 K gluconate (pH adjusted to 7.3 with KOH). Current data was recorded at 10 kHz using a DA/AD interface (Digidata 1322A, Molecular Devices, Sunnyvale, CA, USA). Drugs were applied using a multichannel perfusion system (Model BPS-8, Scientifica, Uckfield, UK) positioned 150 µM away from the recorded cell and controlled by Clampex 9 software (Molecular Devices, Sunnyvale, CA, USA).

### Fluorescence-based Intracellular Calcium Imaging

Fluorescence-based calcium imaging experiments were carried out 6–10 days after plating of cells. Cells were loaded in the dark for 60 min at room temperature (22°C), in HBTS (Invitrogen, Paisley, UK) containing 4 µM of calcium-sensitive dye Fluo-4 AM (Invitrogen,Paisley, UK) in the presence of 1% pluronic acid (Invitrogen, Paisley, UK). Fluo-4 (*K*_d_ for Ca^2+^ = 345 nM) and Fluo-4FF (*K*_d_ for Ca^2+^ = 9.7 µM) were used for experiments where intracellular calcium responses were expected to be relatively weak or strong, respectively. Cells were washed and continually perfused during the experiment with HBTS containing (mM): 135 NaCl, 5 KCl, 1.2 MgCl_2_, 2.5 CaCl_2_, 10 HEPES, 11 glucose, 0.001 TTX, pH = 7.2. The perfusion flow rate was 3 ml/min, which lead to complete replacement of the 100 µl volume in each well every 2 seconds. Dye-loaded cells were viewed using an inverted epifluorescence microscope (Axiovert,135TV, Zeiss, Cambridge, UK). Fluo-4 fluorescence was excited by a 480±10 nm light source (Polychrome II, TILL-Photonics, Gräfelfing, Germany) and emission was captured by an iXon 897 EMCCD camera (Andor Technologies, Belfast, UK) after passage through a dichroic mirror (505LP nm) and a high pass barrier filter (515LP nm). Digitised images were stored and processed by using Imaging Workbench 5.0 software (INDEC Biosystems, Santa Clara, CA, USA). Data were analysed by averaging individual traces collected from a large number of cells in multiple wells of the 96-well plate. Delta F/F0 values were measured by quantifying the ratio between the change in fluorescence signal intensity (delta F) and baseline fluorescence (F0).
